# Dynamic Development of Viral and Bacterial Diversity during Grass Silage Preservation

**DOI:** 10.3390/v15040951

**Published:** 2023-04-12

**Authors:** Johan S. Sáenz, Bibiana Rios-Galicia, Bianca Rehkugler, Jana Seifert

**Affiliations:** 1 Institute of Animal Science, University of Hohenheim, Emil-Wolff-Str. 6-10, 70593 Stuttgart, Germany; 2 HoLMiR—Hohenheim Center for Livestock Microbiome Research, University of Hohenheim, Leonore-Blosser-Reisen Weg 3, 70593 Stuttgart, Germany

**Keywords:** grass silage, phages, metagenomics

## Abstract

Ensilaging is one of the most common feed preservation processes using lactic acid bacteria to stabilize feed and save feed quality. The silage bacterial community is well known but the role of the virome and its relationship with the bacterial community is scarce. In the present study, metagenomics and amplicon sequencing were used to describe the composition of the bacterial and viral community during a 40-day grass silage preservation. During the first two days, we observed a rapid decrease in the pH and a shift in the bacterial and viral composition. The diversity of the dominant virus operational taxonomic units (vOTUs) decreased throughout the preservation. The changes in the bacterial community resembled the predicted putative host of the recovered vOTUs during each sampling time. Only 10% of the total recovered vOTUs clustered with a reference genome. Different antiviral defense mechanisms were found across the recovered metagenome-assembled genomes (MAGs); however, only a history of bacteriophage infection with *Lentilactobacillus* and *Levilactobacillus* was observed. In addition, vOTUs harbored potential auxiliary metabolic genes related to carbohydrate metabolism, organic nitrogen, stress tolerance, and transport. Our data suggest that vOTUs are enriched during grass silage preservation, and they could have a role in the establishment of the bacterial community.

## 1. Introduction

Silage can be defined as feed resulting from forage preservation in a succulent condition, mediated by fermentation in a more-or-less airtight container [1]. Conservation of forage feed by ensilaging is one of the most common fermentation processes in the northern hemisphere. It is estimated that 200 million tons of dry matter are ensiled annually [2]. Ensiled forages are widely used because they reduce nutrient loss, simplify feeding, improve animal/farm management, and may have a probiotic effect [3,4]. Production of high-quality silage depends on several factors such as harvest time, ensiling time, the raw material, packing, use of additives, and the efficiency of the ensiling phases [5,6]. The success of the ensilaging is mainly driven by microorganisms. During the fermentation phase, lactic acid bacteria (LAB) dominate the process due to the decrease of the pH because of acid production [7]. In addition, unwanted microorganisms such as Enterobacteria, yeast, molds, and acetic acid bacteria are inhibited. Several members of the *Lactobacillaceae* family have been reported as key organisms, with *Lactobacillus plantarum* and *Lactobacillus buchneri* being the most recurrent ones [8,9,10].

The microbial succession during ensiling has been focused on the interaction between lactic acid bacteria and unwanted microorganisms, but the role of the virome is still unknown. Due to the recent recognition of the role of bacteriophages as a gut health modulator [11,12], their enumeration and characterization in food or feed fermentation have gained importance. Phages are the most abundant and diverse biological entities on the planet. Their ecological role includes the regulation of bacterial and archaeal diversity, as well as the mobility of genetic material [13]. For that reason, the interest and study of such viruses have greatly increased during the last few years.

Individual bacteriophages have been studied and isolated from silage samples. Their presence has been linked to both loss and gain of silage quality. For example, on the one hand, the presence of *Lactobacillus* phages has been associated with a decrease in the fermentation quality in Japan [14]. On the other hand, ~50% of silage samples from a dairy farm with low *Listeria monocytogenes* positiveness were shown to harbor listeriaphages [15]. Both findings suggest that phages could play a key role during the ensiling process and thus might provide the basis of a biotechnological application aimed at silage conservation.

The role of endogenous bacteria and bacteriophages in silage development is poorly understood. The same is true for the subsequent effect on the animal’s performance and health when feeding ruminants with silages of lowered quality or with a specific load of microbial members. This raises the question about the possible modulatory role of phages on the intersection between diet, microbiome, and gut health. In the frame of this study, we investigated the bacterial and viral community during 40 days of grass preservation. The objective was to describe the dynamics of bacteria and phages and their probable interaction during the fermentation process.

## 2. Materials and Methods

### 2.1. Silage Preparation and Experimental Design

Annual raygrass (*Lolium multiflorum)* mixed with clover, flowers, and herbs typical of a pasture for dairy cows in Germany was cut in August 2021 at Hohenheim University and chopped into smaller pieces using a straw grass grinder. Approximately 600 g of grass was packed in 1 L airtight glass jars (WECK Wehr, Germany). In total, 15 jars were prepared and randomly assigned to 5 sampling times, three jars per time point (2, 4, 8, 15, and 40 days). Each jar was weighed after being sealed and before opening on the respective sample day. Additionally, 500 g of fresh grass was vacuum-packed in plastic bags and frozen at −80 °C. Jars were maintained in the dark at 24 °C until sampling. During each sampling time, three jars were opened and the pH was determined using 25 g of the material. Silage was mixed with 100 mL of distilled water, and after 15 min, the pH was measured using a pH-meter (Mettler Toledo, Gießen, Germany), while stirring the solution [16]. Samples were vacuum-packed in plastic bags and frozen at −80 °C.

### 2.2. DNA Extraction, Sequencing, and qPCR

Briefly, 5 g of the frozen material was mixed with 40 mL of PBS buffer (pH 7.5, 37 mM NaCl, 2.7 mM KCl, 8 mM Na_2_HPO_4_, and 2 mM KH_2_PO_4,_) in a 50 mL Falcon tube. The sample was mixed for 30 min in a horizontal shaker and manually shaken every 10 min. This mixture was filtered using a sterile cheesecloth, transferred to a new Falcon tube, and centrifuged 30 min × 15,557× *g* at 4 °C. The supernatant was carefully discarded, and the pellet was kept. DNA was extracted only from the obtained pellet as follows. The pellet was dried in an inverted position for 5 min. Finally, the pellet was eluted using 800 μL of CD1 solution from the QIAamp PowerFecal pro kit (QIAGEN, Hilden, Germany). DNA was extracted following the instructions of the manufacturer after adding the CD1 solution. The quantity and quality of the DNA were checked using 1% of agarose gel and Nanodrop measurements (Thermo Fisher Scientific, Darmstadt, Germany). Additionally, total DNA was quantified using the QuantiFluor^®^ dsDNA System kit following the manufacturer’s instructions (Promega, Heilbronn, Germany) in a Qubit 4 Fluorometer (Thermo Fisher Scientific, Darmstadt, Germany).

Amplicon sequencing libraries were prepared according to the protocol described by Roth et al. (2022) [17]. Two-step PCR was carried out using PrimeSTAR^®^ HS DNA Polymerase kit (TaKaRa, Beijing, China) and the primers 27F and 338R were used to target the V1–V2 region of the 16S rRNA gene. The first PCR was prepared in a total volume of 25 µL using 1 µL of DNA template, 0.2 µM of each primer, and 0.5 U Taq prime start HS DNA. The second PCR was prepared in a total volume of 50 µL. An initial denaturation at 95 °C for 3 min was followed by ten cycles (first PCR) or 20 cycles (second PCR) of denaturation at 98 °C for 10 s, annealing at 55 °C for 10 s and an extension at 72 °C for 45 s. The final extension was carried on at 72 °C for 2 min. PCR products were purified and standardized using SequalPrep Normalization Kit (Invitrogen Inc., Waltham, MA, USA). Sequencing was carried out using 2 × 250 bp paired-end sequencing chemistry on Illumina Novaseq 6000. Metagenomic Illumina library preparation and sequencing of 2 × 150 bp reads in a Novaseq 6000 (Illumina, Inc., San Diego, CA, USA) were carried out by Novogen. In short, the genomic DNA was randomly fragmented by sonication, then DNA fragments were end polished, A-tailed, and ligated with the full-length adapters of Illumina sequencing, and followed by further PCR amplification with P5 and indexed P7 oligos. The PCR products as the final construction of the libraries were purified with AMPure XP system. Then, libraries were checked for size distribution by Agilent 2100 Bioanalyzer (Agilent Technologies, Santa Clara, CA, USA), and quantified by real-time PCR (to meet the criteria of 3 nM).

Quantification of total and lactic acid bacteria was carried out by qPCR using the CFX Connect™ Real-Time PCR Detection System (BioRad Laboratories Inc., Feldkirchen, Germany). The PCR was prepared in a total volume of 20 μL, where 1 μL of each DNA extraction was mixed with 10 μL of GoTaq 2× qPCR Master Mix (Promega, Heilbronn, Germany) and 0.2 uM of each primer. For total bacteria, the primers BAC338F (5′-ACTCCTACGGGAGGCAG-3′) and BAC805R (5′-GACTACCAGGGTATCTAATCC-3′) were used [18], while for the total lactic acid bacteria 5′-AGCAGTAGGGAATCTTCCA-3′ and 5′-CACCGCTACACATGGAG-3′ were used [19]. Details of qPCR cycles and standard curve preparation can be found in Supplementary Material S1.

### 2.3. Prokaryotic Community, Binning, Taxonomic, and Functional Annotation

Raw amplicon sequencing data were demultiplexed using Sabre (https://github.com/najoshi/sabre) and processed by QIIME2 v.2021.2-8 [20]. Briefly, primers were removed using q2-cutadapt [21] and the reads were denoised with q2-dada2 [22]. Obtained amplicon sequence variants (ASVs) were taxonomically assigned based on the consensus between VSEARCH [23] and prefitted sklearn classifiers [24] against the Silva SSU-rRNA database v.138.1 [25]. The Silva database was downloaded and curated using RESCRIPt [26]. The alfa and beta diversity were calculated using QIIME. The richness (number of ASVs) and Shannon diversity (accounts for both abundance and evenness) indexes were used to describe the alfa diversity. Unweighted UniFrac distance metric was used to calculate the dissimilarity between pairs of samples, and a principal coordinates analysis (PCoA) was used to visualize the dissimilarities.

Metagenomic raw reads, ~100 Gb of data, were quality-controlled using BBduck v38.26 (https://sourceforge.net/projects/bbmap/). Sequencing artefacts, adapters, and short reads were removed. After that, reads were taxonomically annotated using Kaiju v1.9.0 [27] and the NCBI RefSeq database (Archaea, Bacteria, and viruses). MetaSPADES v3.15 [28] was used to assemble clean reads, and the resulting contigs longer than 1000 bp were kept. Bins were binned using Metabat2 [29], Maxbin2 [30] and Concoct [31], all tools wrapped in MetaWRAP [32]. Obtained bins were refined and aggregated using MetaWRAP. CheckM [33] was used to estimate the quality of the bins, and the mid- to high-quality bins with >70% completeness and <10% contamination were kept for further analysis. Metagenome-assembled genomes (MAGs) were taxonomically classified using GTDB-tk v2.1 [34] and the GTDB database R207_v2 [35]. Antiviral defense mechanisms were identified from all MAGs using PADLOC v1.1 [36] and the PADLOC-DB. CRISPR-Cas genes and arrays were also detected using CRISPRCasTyper v1.6 [37]. Obtained spacers were extracted and blasted against contigs identified as vOTUs, and only those with >95% identity and <0.0004 e-value were considered spacer to protospacer matches. MAGs were dereplicated at 99% ANI (ANImf) using dRep [38], including quality information obtained with checkM. CoverM (https://github.com/wwood/CoverM) was used to calculate the relative abundance of dereplicated MAGs across all samples mapping the clean reads against the MAGs. Further, metabolic traits of MAGs were determined with DRAM [39]. Finally, dereplicate MAGs were phylogenetically placed using Phylophlan [40] and 400 universal marker genes, with medium diversity and accurate options.

### 2.4. Comparison of Lactococcus *sp.* (C4C_bin8) Defense Mechanism

The MAG identified as *Lactococcus* sp. (C4C_bin8) was genomically compared against 19 *Lactococcus* isolates (Supplementary Material S2). All genomes were collected from the NCBI gene bank and pretreated to discard contigs shorter than 2.5 kb. The core genome calculation was performed considering the presence of single-copy genes in at least 90% of the genomes and an index of geometric homogeneity of 0.95. Additionally, the relatedness of the C4C_bin8 MAG with other *Lactococcus* was calculated using taxonomic pairwise indices of average nucleotide identity (ANI) [41], average amino acid identity (AAI), core-gene average amino acid identity (cAAI) [42], and percentage of conserved proteins (POCP) [43]. The scores were calculated using pyANI v0.2 in Anvi’o [44], ezAAI v1.2.1 [45], and POCP v1.1.1 (https://github.com/hoelzer/pocp). Defense mechanisms from all *Lactococcus* species were annotated using PADLOC v1.1 and PADLOC-DB.

### 2.5. Viral Prediction, Taxonomic, and Functional Annotation

Assembled metagenomes were searched for viral contigs using Virsorter2 v2.2.3 [46] and VIBRANT v1.2.1 [47]. Predicted viral contigs, >10,000 bp by both tools, were merged and dereplicated using dRep at 95% identity and 85% coverage (dRep dereplicate -d -p 25 --S_algorithm ANImf -nc .85 -l 10000 -N50W 0 -sizeW 1 –ignoreGenomeQuality –clusterAlg single). Dereplicated viral contigs, representative of the vOTUs, were quality-checked using checkV [48]. Dereplicated vOTUs with 0 viral genes and >1 host gene were discarded. vOTUs with 0 viral genes and 0 host genes were kept. A vOTU was considered provirus or complete if it was predicted as such by checkV. Coverage of vOTUs, as reads per kilobase of exon per million reads mapped (RPKM) across samples, was calculated using coverM with “contig” mode. The vOTUs were taxonomically classified with PhaGCN2 [49] based on the latest International Committee on Taxonomy of Viruses (ICTV) classification (https://ictv.global/). Obtained viral families classified as “Family_like” were renamed as “Other”. Phage host prediction was carried out with iPHhoP (https://bitbucket.org/srouxjgi/iphop) and its default database. Auxiliary metabolic genes (AMGs) were searched across the vOTUs using DRAM-v [39] with the UniRef90 database and other default databases but without the KEGG database. For DRAM output, only putative AMGs were retained; no viral flag (F), transposon flag (T), viral-like peptidase (P), or attachment flag (A) could be present, and putative AMGs that did not have a gene ID or a gene description were also discarded.

### 2.6. Clustering, Taxonomic Context and Phylogenetic Analysis of vOTUs

vConTACT2 [50] was used to cluster the vOTUs against 17,278 bacteriophage genomes from the Genbank. Genomes were obtained from the 2Nov2022 dataset created with Iphared [51]. Proteomes of the vOTUs were predicted with Prodigal [52] and used to phylogenetically place them using VICTOR [53]. Taxon boundaries at the species, genus, and family level were estimated with OPTSIL [54]. Tree was inferred using the formula D6 yielded average support of 57% and was visualized using iTol (https://itol.embl.de/) accessed on 20 September 2022.

### 2.7. Data Wrangling and Data Availability

All data wrangling and statistical analyses were performed in R v4.1.2 and the packages “tidyverse”, “data.table”, “ggplot”, “patchwork”, “ggtext”, “vegan”, “ape”, and “viridis” were used. The data processing workflow can be found as a Jupyter-lab notebook at https://github.com/SebasSaenz/silage_timetrial, as well as other R scripts for data wrangling and visualization. Raw sequences and MAGs were deposited in the European Nucleotide Archive (ENA) under the Bioproject PRJEB59010.

## 3. Results

### 3.1. Silage Characterization

**Figure 1:**
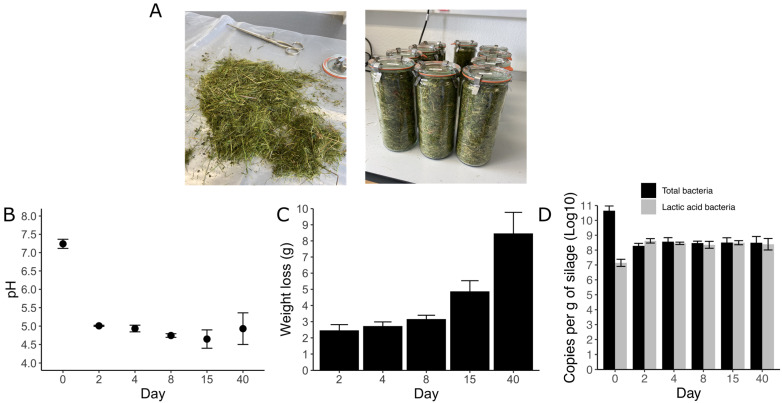
Characterization of a 40-day grass ensilaging process. The grass was stored in (**A**) 1 L glass jars, and during each sampling time the (**B**) pH, (**C**) weight loss, and (**D**) 16S rRNA gene copy number (log10 scale) of total bacteria and lactic acid bacteria per gram of silage were measured.

During each sampling time (0, 2, 4, 8, 15, and 40 days), the grass was characterized by measuring the pH, weight loss, and the copy numbers of total bacteria and lactic acid bacteria (Figure 1). Before ensilaging, the pH of the grass was ~7 and, as expected, declined rapidly within the first two days. The pH oscillated between 4.5 and 5.28 and remained stable until the last sampling day, indicating the probable production of different acid products. The weight loss did not show a rapid trend such as pH. Comparing the first eight days after ensilaging, the mass loss was not significant, but after 15 and 40 days, approximately 5 and 9 g were lost per jar (Figure 1B). A rapid shift in the abundance, measured by qPCR, between total bacteria and lactic acid bacteria was also notable. Within the first two days, lactic acid bacteria represented ~98% of the total bacteria, while it was ~60% before the ensilaging (Figure 1D).

### 3.2. Prokaryotic Community

**Figure 2:**
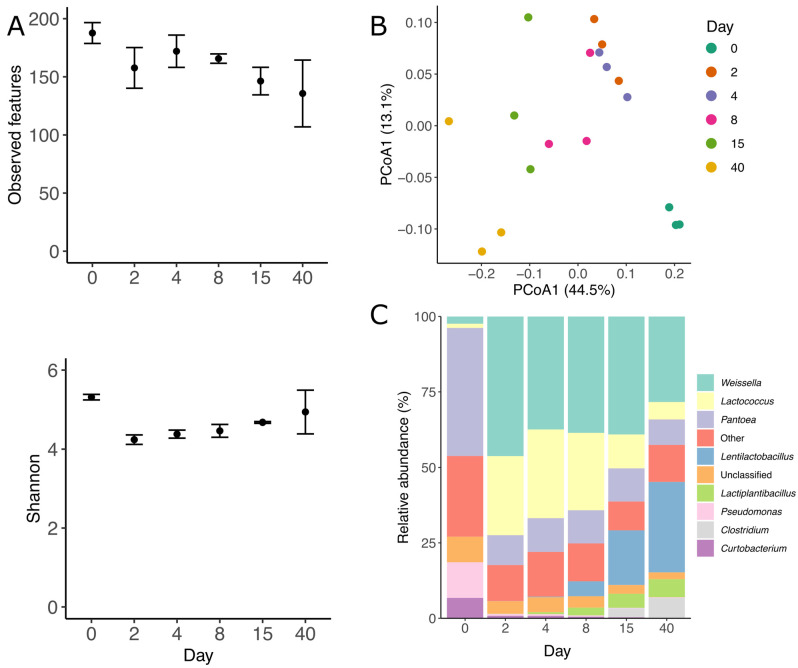
Bacterial diversity and abundance during grass ensilaging based on 16s rRNA amplicons. (**A**) Observed features (ASVs) and Shannon diversity, (**B**) ordination plot (PCoA) of bacterial ASVs based on unweighted UniFrac distances. Each circle represents a sample, and the color represents the time the sample was taken. The closer the circles, the more similar the bacterial communities are. The number between parenthesis indicates the variance explain in each axis. (**C**) Bacterial taxonomy at the genus level across 40 days of ensilaging. Others represent all genus < 5% abundant.

In total, 18 amplicon sequencing libraries spanning the V1–V2 region of the 16S rRNA gene were used to analyze the bacterial community. Amplicon sequencing showed that grass ensilaging changed rapidly the bacterial structure and composition (*p* = 0.001, PERMANOVA, Figure 2B). The ordination plot shows that the biggest differences in ASVs composition can be found between the bacterial communities of day 0 and 40. Across all samples, 323 different ASVs were found, with the pre-ensilaging time being the one with the highest richness. Additionally, the number of observed ASVs significantly decreased during the 40 days of fermentation (*p* = 0.02, Kruskal–Wallis, all groups), while the diversity (Shannon diversity) decreased after the second day but remained stable the rest of the time (*p* = 0.03, Kruskal–Wallis, all groups, Figure 2A). The bacterial community was mainly dominated by members of three phyla, *Firmicutes*, *Proteobacteria*, and *Actinobacteria*, which represented, on average, 66, 30, and 2.8% of the total obtained sequences across all sampling points. The bacterial community rapidly shifted between days 0 and 2, *Firmicutes* increased from 8 to 75%, *Proteobacteria* decreased from 79 to 22%, and Actinobacteria decreased from 11 to 2%. After 40 days, *Firmicutes* dominated the community, reaching 84%, while *Proteobacteria* and *Actinobacteria* decreased to 15 and 0.5%, respectively. On the fresh grass (day 0), *Pantoea*, *Pseudomonas* and *Curtobacterium* were the most abundant genera, while *Weissella*, *Lentilactobacillus*, *Pantoea*, and *Lactiplantibacillus* were the dominant ones at the end of the preservation (Figure 2C). A similar bacterial structure and composition were observed with the metagenomic sequencing data (Figure S1).

### 3.3. Metagenomic Binning Reveals the Potential for Antiviral Defense in Silage

**Figure 3:**
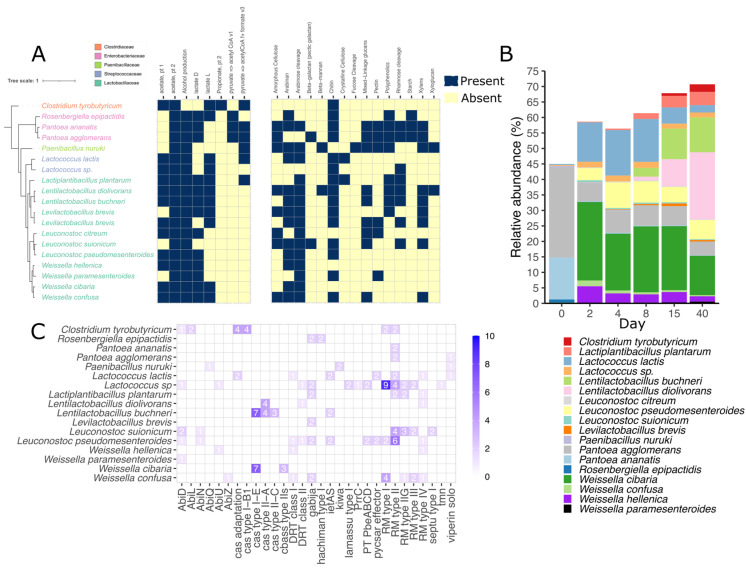
Functional potential of the most abundant bacterial members (MAGs) during grass ensilaging. The tree was constructed based on 400 universal marker genes and rooted at the *C. tyrobutyricum* node. (**A**) Metabolism is grouped by SCFA and alcohol conversion and carbohydrate-active enzyme targets. (**B**) Relative abundance of the 19 recovered MAGs and (**C**) antiviral defense mechanism found in them. The numbers indicate the number of genes.

Genome-resolved metagenomics was applied to three fresh-cut grass (day 0) and 15 silage samples. Samples were assembled and binned individually, and obtained bins were dereplicated based on their similarity and quality. In total, we binned 123 medium- to high-quality draft metagenome-assembled genomes (MAGs). After dereplication at 99% ANI, only 19 MAGs were kept as strain representatives, of which 9 were considered high-quality drafts (>90% completeness and <5% contamination) and 10 medium-quality drafts (>75% completeness and <10% contamination). In average, dereplicated MAGs had an average N50 of 54,972 bp and N75 of 25,234 bp. Most of the MAGs, 12 of them, belonged to the *Lactobacillaceae* family, while three to *Enterobacteriaceae*, two to *Streptococcaceae*, one to *Paenibacillaceae*, and one to *Clostridiaceae*. We mapped the raw reads against the recovered MAGs and estimated that they represented around 45–70% of the microbial community, with the lowest values at day 0 and the highest at day 40 of ensilaging (Figure 3B). The genomes were differentially abundant across the pre- and ensilaging days. For instance, MAGs identified as *Pantoea* comprised almost 40% of the total microbial community in the fresh grass, while they decreased to ~4.5–7.3% after the second day of ensilaging. A succession between *Lactococcus lactis* and different members of the *Lactobacillaceae* family was evident between days 8 and 15 of ensilaging. Interestingly, the relative abundance of *Leuconostoc pseudomesenteroides*, *Pantoea agglomerans*, *Weissella cibaria*, and *Weissella paramesenteroides* remained stable within the second and 40th days of the experiment. *Clostridium tyrobutiricum* was the only genome not belonging to *Lactobacillaceae* that increased its abundance between the last days.

The functional potential of the recovered MAGs was diverse. Almost all genomes harbor the potential to produce alcohol, acetate, and lactate, while only *C. tyrobutiricum* seems to produce propionate. Likewise, 31% of the taxa can produce formate via pyruvate (Figure 3A). In addition, four types of carbohydrate-active enzymes (CAZymes) involved in the synthesis, metabolism, and recognition of complex carbohydrates were detected. Those enzymes were glycoside hydrolases (GHs: 41), polysaccharide lyases (PLs: 5), carbohydrate esterases (CE: 4), and auxiliary activity (AA: 4). The most prevalent enzymes found across the genomes were alpha-amylase (GH13), beta-glucuronidase (GH1), and lysozyme (GH73). After data distillation using DRAM, most of the genomes seemed to have the potential of degrading chitin, arabinose, and arabinan. Interestingly, the potential to degrade more complex structures was found in fewer genomes. For example, only two taxa seemed to degrade crystal cellulose (*L. lactis* and *Lactiplatibacillus plantarum*), while two different taxa could hydrolyze beta-mannan using the enzyme CE2 acetyl xylan esterase (*Paenibacillus nuruki* and *Lentilactobacillus diolivorans*). All CAZymes found in the recovered MAGs are described in detail in the Supplementary Material S2.

Several defense mechanisms and secondary metabolism-related genes were found across the recovered MAGs. PADLOC was used to search the proteomes of all 123 recovered genomes and identify antiviral defense mechanisms. In total, 29 different defense systems were found in 17 different species (Figure 3C). The most common system was the restriction modification (RM) type II, RM type IV, and Gabija systems present in 42%, 32%, and 32% of the total species. One MAG (C8A.bin.4) identified as *Lactococcus* sp. harbored 11 different defense systems, being the most in all recovered genomes. *L. citreum* was the only species not carrying any defense mechanism. In addition, cas-type genes were rarely found in the genomes and were only present in ~26% of them. On the other hand, antiSMASH was used to predict biosynthetic gene clusters (BGC). Altogether, 13 different BGCs were predicted by antiSMASH in 15 out of 19 searched species (Figure S2). The three main BGCS were Type III Polyketide Synthases (T3PKS), Ribosomally synthesized and post-translationally modified peptides (RIPP), and Terpene, comprising 58% of all hits. *P. agglomerans* was the species with the most total BGCs and different BGCs, 7 and 6, respectively, followed by *C. tyrobutiricum* and *Leuconostoc suionicum*. *Lactococcus* sp. harbored only one RIPP-like related cluster.

### 3.4. Lactococcus Species Encodes a Variety of Defense Mechanisms in Silage

Two MAGs (C8A.bin.4 and C4C.bin.8) identified as *Lactococcus* sp., harbored the greatest number of defense mechanism among the assembled genomes. However, as the species could not be identified, the C4C.bin.8 assembly was compared against 18 *Lactococcus* species from different origins. The analysis grouped the MAG C4C.bin.8 among a cluster of Lactococci strains originating from humans and fermented food (*L. garvieae*, *L. petauri*, and *L. formosensis*) (Figure S3, Supplementary Material S2). Among this group, ANI, AAI, and cAAI indices related with higher scores (92–97%) than the rest of Lactococci (70–61%). POCP scores, however, differed among the aforementioned group, C4C.bin.8 shared higher scores with *L. garvieae*, *L. taiwanensis*, *L. allomyrinae*, and *L. protatiae* (54–41%) than with the rest of Lactococci (<35%). Pairwise indices for C4C.bin.8 were on the threshold for ANI percentage, higher than the threshold for cAAI, and below the threshold for AAI. In general, the *Lactococcus* genomes harbored four different types of defense system, while the two MAGs from silage harbored 11 and seven, respectively. The most common systems were RM type I and II; however, the MAGs also additionally carry the type IIG, and III. Other defense mechanisms, such as tmn, PrrC, and lamasu type I (Figure S4), were found exclusively in the silage *Lactococcus* sp. (C8A.bin.4 and C4C.bin.8).

### 3.5. Viral Diversity during Grass Ensilaging

Using Virsorter and VIBRANT, we predicted viral scaffolds from 18 metagenomic samples collected at different times during grass ensilaging. Three of the samples represented the pre-ensilaging (day 0), and the remaining samples had different sampling times across 40 days. In total, 2348 and 10,005 of 633,828 scaffolds were predicted as viral by Virsorter and VIBRANT, respectively. After dereplication (95% identity and 85% coverage) and quality control, we identified 240 viral scaffolds longer than 10,000 bp. These viral scaffolds were considered viral operational taxonomic units (vOTUs). Based on the Minimum Information about an Uncultivated Virus Genome (MIUViG) standards, 227 of the vOTU were genome fragments while 13 vOTU were high-quality draft genomes. The average length of the predicted vOTU was 17.6 ± 8.7 kbp, with the longest being 64 kbp and the smallest being 10 kbp (length threshold). The average length of vOTU classified as proviruses was 28.2 ± 10.6 kbp. On average, 10.5 ± 7.8 viral genes were predicted per genome, with 39 genes being the maximum found. According to CheckV, the viral draft genomes varied in quality: 0.8% “complete”, 4.6% “high quality”, 13.0% “medium quality”, 78.0% “low quality”, and 3.8% “not determined”. From these 240 vOTUs, 23 were predicted to be proviruses. An overview of the vOTUs genomes can be found in Supplementary Material S2.

**Figure 4:**
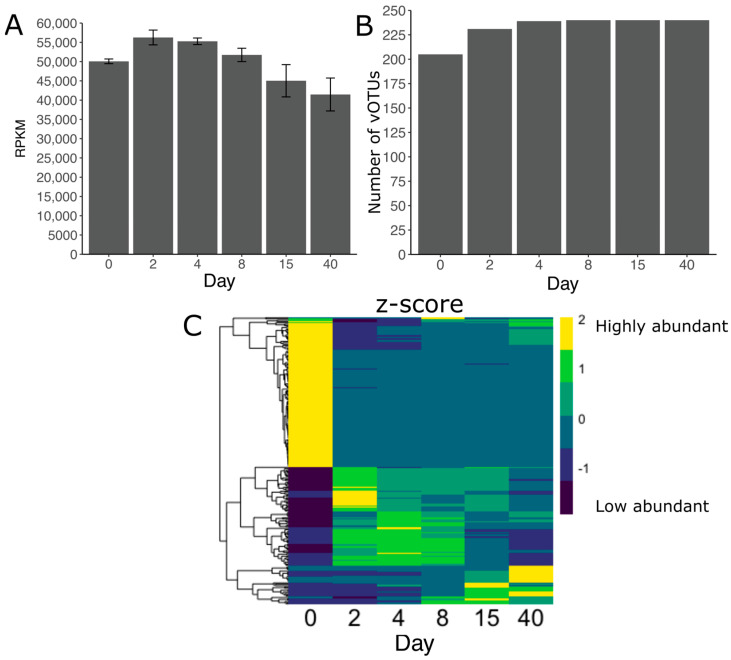
Abundance and diversity of 240 vOTUs recovered from grass ensilaging. (**A**) Total abundance of the vOTUs presented as reads mapped per kilobase of contig, per million mapped reads (RPKM). (**B**) Number of vOTUs found per day. (**C**) Differential abundance (z-score) of the vOTUs across sampling time. The heatmap clustering is based on abundance patterns. The vOTUs identified as provirus are not shown in the heatmap.

We found a large degree of heterogeneity in the vOTUs between samples, in terms of relative abundance and number of observed vOTUs, which formed two clusters based on the degree and direction of change in relative abundance (Figure 4C). During the first two days, a higher diversity was present in the vOTUs that dominated the community (cluster one); however, after ensilaging, an enrichment of fewer populations was evident (cluster two). This trend repeated throughout the whole experiment and with different vOTUs at each sampling time (Figure 4C). The vOTUs reached their maximum number only after day 8 of the ensilaging (Figure 4B). Additionally, the relative abundance of the whole population was highly variable, with an increase within the first 4 days but a decrease after day 8. We also observed a succession between the vOTUs in terms of differential abundance.

**Figure 5:**
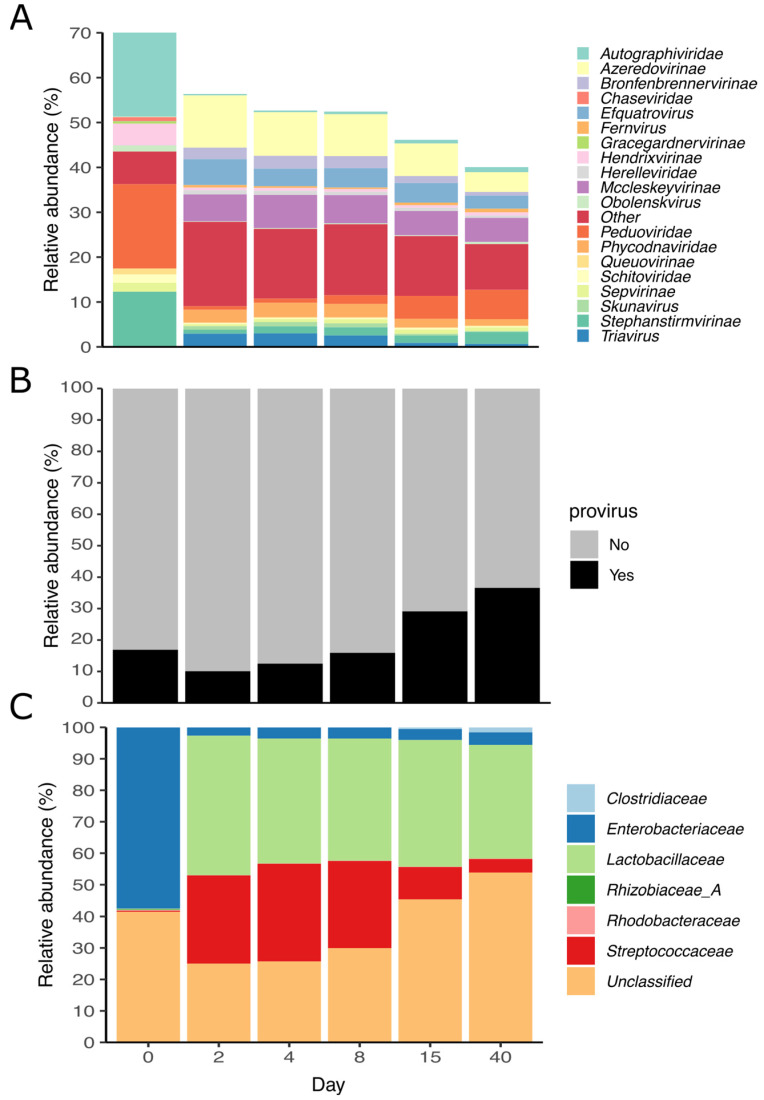
Characterization of the vOTUs during ensilaging. (**A**) The relative abundance of viral families, (**B**) proviruses, and (**C**) viral host during the ensilaging are shown. The relative abundance of 240 vOTUs was calculated based on the reads mapped per kilobase of contig per million mapped reads (RPKM) across each sample.

Altogether, 71% of the vOTUs were taxonomically annotated at the family level using the latest ICTV classification. Around 21% of annotated populations were labeled as “family_like” by PhaGCN, indicating a similar but unknown family. On average, the most abundant families across all samples were *Azeredovirinae* (7.1%), *Peduoviridae* (5.7%), *Mccleskeyvirinae* (3.7%), and *Autographiviridae* (3.5%). Before ensilaging, the families *Peduoviridae*, *Autographiviridae*, and *Stephanstirmvirinae* were the most abundant ones, while after the ensilaging, *Azeredovirinae*, *Mccleskeyvirinae*, and *Efquatrovirus* dominated the community (Figure 5A). Interestingly, the relative abundance of *Peduoviridae* members decreased up to 0.7% but increased again up to 6.5% within 15 and 40 days of ensilaging. On average, the relative abundance of proviruses across all samples was 20.2%, with an increase between days 2 and 40 and a peak of 36.5% at the end of the ensilaging (Figure 5B). Additionally, we predicted the putative host of 63% of the vOTUs. The relative abundance of the putative host resembled the bacterial community observed with the amplicon and metagenomic data (Figure 5C). The vOTUs infecting *Enterobacteriaceae* were more abundant before ensilaging, while vOTUs infecting *Streptococcaceae* and *Lactobacillaceae* increased within the first two days. This reassembled the two clusters observed in Figure 5C, where vOTUs with *Enterobacteriaceae* as putative hosts belong to the highly abundant cluster before the ensilaging. The vOTUs infecting *Streptococcaceae* decreased within days 8 and 40, and no such trend was observed for *Lactobacillaceae*. It is important to note that the most abundant vOTUs after 40 days of ensilaging were often unclassified or not linked to a probable host.

**Figure 6:**
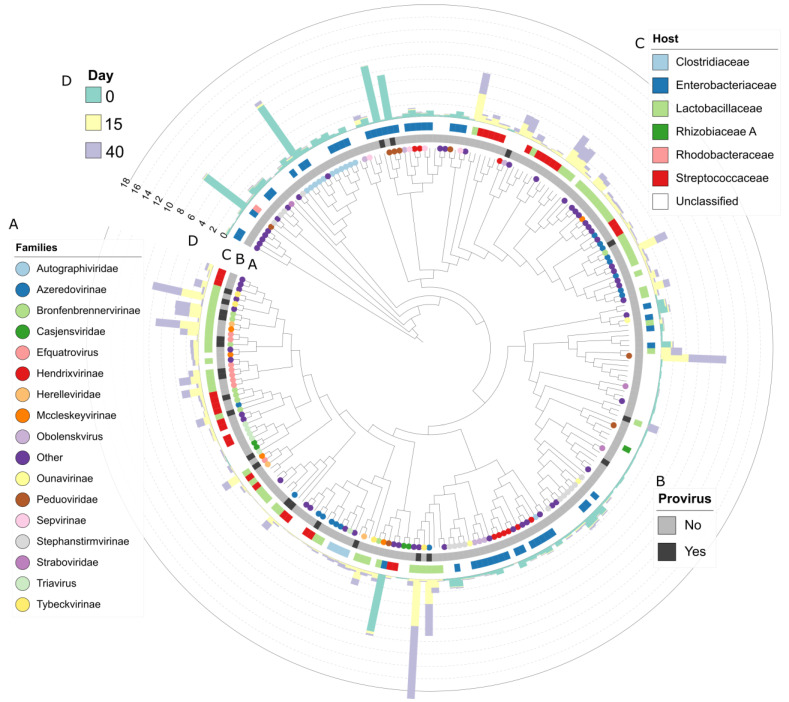
Phylogenetic relationship of 240 vOTUs recovered during 40 days of grass ensilaging and their abundance across time. The plot displays the changes of the relative abundance of the different vOTU across time, their taxonomic classification and potential host. The tree was constructed using the predicted proteins of each vOTU and the Virus Classification and Tree Building Online Resource (VICTOR). Each branch represents a unique vOTU (240 total). The colored circles indicate the viral family (**A**), grey/black squares (**B**) if they are provirus, colored squares are the predicted host (**C**), and the bar plot (**D**) shows the relative abundance of each vOTU at 0 (green), 15 (yellow), and 40 (purple) days.

We used VICTOR and OPTSIL to produce a genome-based phylogeny and classification of the vOTUs. The OPTSIL method clustering yielded 46 family clusters, 121 subfamily clusters, 167 genus clusters, and 239 species clusters (Figure 6). The last indicated that of all recovered vOTUs, only two belong to the same species. Additionally, Figure 6 shows that vOTUs enriched in different days are closely related and shared similar hosts. vConTACT2 was used to cluster the grass silage viral genomes with 17,278 known prokaryotic virus genomes from the NCBI at the genus level. In total, 112 vOTUs clustered among themselves or with the reference genomes, while 45 vOTUs were classified as “singletons” and 83 as “outlier”. Singleton vOTUs shared few or no proteins with the reference genomes or other vOTUs, while outliers could be associated with existing proteins but without confidence. vOTUs from the silage tended to cluster among themselves, forming groups of two to five vOTUs. Only 26 of the total vOTUs clustered with a reference genome. This indicates that the majority of the recovered vOTUs from grass silage belong to a novel taxa.

### 3.6. Acquired Immunity and Host-Phage Interaction

Using CRISPRCasTyper, CRISPR-Cas genes and arrays were searched in the 123 recovered medium- to high-quality draft MAGs (no dereplicated). In summary, a complete CRISPR-Cas loci was found in 17 MAGs, while the presence of spacers (1158) was observed in 27 MAGs. Complete CRISPR-Cas were only found in members of the *Lactobacillaceae* family, *W. cibaria*, *L. buchneri*, *L. diolivorans*, and *L. brevis.* In total, 22 arrays were found, 63% belonging to type II-A and 37% to type I-E. In general, one array was found per genome but two arrays were found in five genomes identified as *L. buchneri*. The extracted spacers were blasted against the vOTUs in search of a spacer–protospacer match as an indicator of phage–host interaction. We identified unique interactions between 12 *Lactobacillaceae* MAGs with spacers and three vOTUs (Figure S5). MAGs identified as *L. diolivorans* seem to have an interaction history with vOTUs classified at the family level as *Azeredovirinae* and *Tybeckvirinae*, while *L. brevis* was with *Drexlerviridae*.

### 3.7. Auxiliary Metabolic Genes (AMG) Found in vOTUs

**Figure 7:**
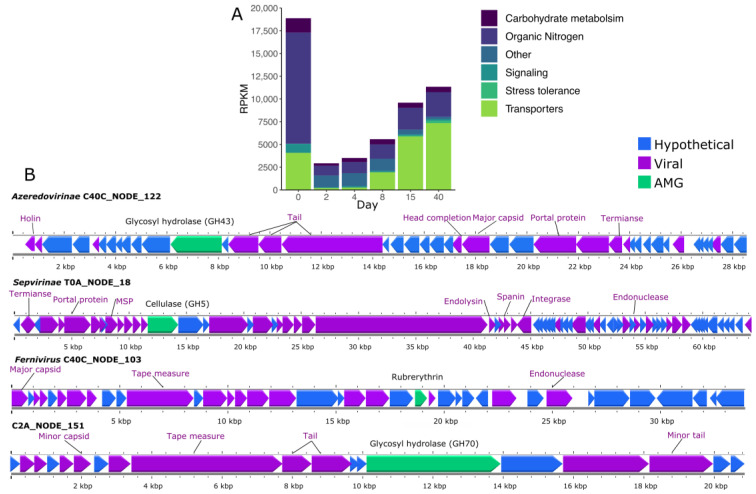
Potential auxiliary metabolic genes (AMGs) found in vOTUs during grass ensilaging. (**A**) Relative abundance of AMGs present in 11 vOTUs grouped by categories. (**B**) Examples of the genomic neighborhood of AMGs found in vOTUs during grass ensilaging. Known viral genes are colored purple, while AMGs and hypothetical genes are green and blue, respectively. Structural viral genes were annotated based on Virus Orthologous Groups (VOGDB) predictions. All genes were annotated using DRAM, and AMGs with flags related to viral (F), transposon (T), viral-like peptidase (P), or attachment flag (A) were discarded.

Across the 240 vOTUs, we identified and annotated 5920 proteins using DRAM. Thirty-six percent of these proteins could be associated with the sequences in the searched database, of which approximately 9% were “uncharacterized” and “hypothetical” proteins. We detected 20 potential auxiliary metabolic genes related to carbohydrate metabolism, organic nitrogen, signaling, stress tolerance, and others across 11 vOTUs. For example, we found a few glycosyl hydrolases (GH70, GH43, and GH5) in three viral genomes recovered at different sampling times (day 0, 2, and 40). GDP-mannose 4,6 dehydratase, another gene associated with carbohydrate metabolism, was found only in one genome. Interestingly, one genome recovered from day 40 harbored the gene rubrerythrin, which is associated with a response to oxidative stress. Rubrerythrin was the only stress-tolerance-related gene found among the vOTUs. Auxiliary metabolic genes were present across all sampling times (Figure 7A), with day 0 exhibiting the highest abundance of viruses carrying these genes and, more specifically, organic-nitrogen-associated genes.

## 4. Discussion

The production and conservation of silage have been under constant progress due to the importance to tackle deficits in feed availability [55]. Most of the progress in silage conservation has derived from studying the role of lactic acid bacteria (LAB) during and after the fermentation phase; however, there are other members of the microbial community that have been underplayed. Recent sequencing and bioinformatic developments have pushed forward the description of complex viral communities and their interaction with other microorganisms [56]. Even though the grass ensilaging process harbors a dynamic, diverse, and unknown virome, this one is underestimated. Here, we used metagenomes and 16S rRNA amplicon libraries to describe the composition and structure of the bacterial and viral community during a 40-day grass ensilaging process. Our study reveals part of the silage taxonomic diversity, its dynamics during the fermentation, and the possible interactions with key members of the ensilaging process. Additionally, found phages carry different potential auxiliary metabolic genes.

Previous phage-silage-related studies are scarce, and they focus on the isolation and individual characterization of mainly enterophages and some LAB phages [14,15,57]. To the best of our knowledge, this is the first metagenomic study to describe the viral diversity during ensilaging. We observed that the grass silage microbiome harbors an unknown and diverse virome. We recovered 240 vOTUs, of which approximately 89% could not be matched with any phage genome in the RefSeq, 29% were not clustered with any known family, and only two of those populations belong to the same species. This shows that most of the silage viral diversity still needs to be discovered. However, it is a common trend that metagenomic studies describe significant portions of novel viruses, as phages are presumed to be the most diverse and abundant lifeforms [13,58]. For example, microbiome studies of products such as cheese [59], kimchi, and vinegar [60] have shown that viral diversity and its role during fermentation is poorly understood and described. In the case of the cheese, similarly to what we found in the grass silage, less than 3% of recovered viral contigs were known phages. This points out that even well-studied industrial processes lack information about their viromes. It is relevant to note that genome-phages databases are vastly incomplete, which limits the classification of phages at different taxonomic levels. Additionally, the recent changes in virus taxonomy ratified by the ICTV [61] removed families such as *Myoviridae* and *Siphoviridae*, which were found to be highly abundant in fermented products such as milk [62] and cheese [59]. Because of that, the comparison of viral taxonomy with previous studies has increased in difficulty.

In total, 29 different viral families were found in the silage and they were differentially abundant before and after the ensilaging. Phage diversity and abundance is affected by several factors such as the phage–bacteria interaction [63], pH [64], temperature, and salinity [65]. In the case of the silage, the bacterial community, pH, and oxygen availability change rapidly after the ensilaging and could be drivers of the phage diversity. The changes in the silage bacterial community resembled the predicted putative host of the recovered vOTUs during each sampling time. That could indicate that the phage–bacteria interaction in silage can be a main driver of phage diversity. In the silage, two groups of phages seem to dominate the community at different times. We observed that the enterophages are most abundant and diverse during the pre-ensilaging and their abundance decreases a few days after ensilaging, whereas phages associated with *Lactobacillaceae* and *Streptococcaceae* increase after the second day of ensilaging. Members of the *Peduoviridae* and *Autographiviridae* families seem to prefer *Enterobacteriaceae* as their host while *Azeredovirinae*, *Mccleskeyvirinae*, and *Efquatrovirus* seem to prefer *Lactobacillaceae.* In other fermented products such as cider [66] and sauerkraut [64], it has been seen that phages can survive strong pH and temperature changes. We can hypothesize that those factors during the ensilaging could have less influence than the bacterial–phage interaction on the phage diversity.

The panimmunity in the grass silage seems to be mainly driven by innate immune mechanisms. Similar observations have been made in cheese-associated bacterial communities [67], where CRISPR does not provide complete immunity against all phages; instead, the innate immune mechanisms may play a complementary role. We found that RM systems were the most prevalent defense mechanism across the recovered genomes from the silage, while CRISPR-Cas arrays (acquire immunity) were not common. Systematic analysis of 21,000 bacterial and archaeal genomes have found that RM systems are, by far, the most abundant mechanism, followed by CRISPR-Cas [68]. CRISPR-Cas are more commonly found in LAB-like lactobacilli members than in general bacteria [69]. Despite most MAGs recovered from silage being LAB, only 13% harbored a complete CRISPR-Cas array. This can suggest that in silage, innate immunity plays a more important role against phages than acquired immunity. However, the prediction of complete arrays in highly fragmented MAGs is challenging and could lead to errors. Interestingly, CRISPR spacers extracted from arrays matched with two different vOTUs in the silage, which shows an infection history and previous bacteria–phage interaction. *L. diolivorans*, harboring CRISPR-Cas array, was the most abundant bacteria after 40 days of ensilaging, which could also indicate that acquired immunity could play an important role in the establishment of the key members of the silage microbiome.

AMGs encoded in the viral contigs were associated with carbohydrate metabolism, organic nitrogen, signaling, and stress tolerance. These results are consistent with previously reported glycosyl hydrolase and stress tolerance genes encoded in viromes from different environments [70,71,72]. The potential transfer of glycosyl hydrolase between different members of the silage microbiome could enhance the degradation of complex carbon structures. Additionally, as silage is used to feed animals, the exchange of such genes could relax the metabolic bottlenecks in the microbial host [73]. However, no evidence of such exchange has been found, and the presence of AMG is not an indication of actual gene mobilization between phage and the host. Some of the AMGs can be misidentified because phages also contain several metabolic-like genes.

Our data suggest that the grass silage viral diversity varies through time, and it seems to be tightly linked to the bacterial community. As silage could potentially be a source of phages with medical and biotechnological applications, it seems relevant to study the dynamic of phages not only in grass silage but also in other plant materials such as corn, alfalfa, or sorghum, as well as all the factors that affect the ensilaging process. This information could lead not only to the isolation of novel species but also the understanding of bacteria–phage interaction in medium-complexity microbial communities.

## 5. Conclusions

This study highlighted the microbial diversity and heterogeneity across 40 days of grass silage fermentation. The vOTUs detected in the metagenomic dataset, and the prediction of their putative host, suggest that multiple interactions between the bacteria and bacteriophages occur during the ensilaging process. The phages could play an equal, or more important, role than the frequently studied unwanted microorganisms in fermented feed, which points to the importance of the characterization of novel diversity in such products.

## Data Availability

Data processing workflow can be found as a Jupyter-lab notebook at https://github.com/SebasSaenz/silage_timetrial. Raw sequences and MAGs were deposited in the European Nucleotide Archive (ENA) under the Bioproject PRJEB59010.

## Supplementary Materials

The following supporting information can be downloaded at: https://www.mdpi.com/article/10.3390/v15040951/s1. Supplementary Material S1: Figure S1. Relative abundance of the most abundant bacterial phyla; Figure S2. Biosynthetic gene clusters (BGCs) found in the recovered MAGs; Figure S3. Taxonomic analysis of the MAG C4C.bin.8 using 400 universal bacterial markers; Figure S4. Antiviral defense system found in a set of Lactococcus complete genomes isolated from different sources and 2 MAGs recovered from grass ensilaging samples; Figure S5. Host virus interaction between bacterial genomes and vOTUs during 40 days of grass ensilaging. Supplementary Material S2: Table S1. MAGs assembly quality; Table S2 MAGs taxonomy; Table S3. MAGs CAZymes; Table S4. Lactococcus genomes NCBI metadata; Table S5. Lactococcus genome comparison indexes; Table S6. vOTUs description; Table S7. vOTUs taxonomy; Table S8. vOTUs putative host; Table S9. vOTUs gene annotation; Table S10. Accession numbers for each biosample archive in the ENA.
