# Supplementary material for: Dynamic Development of Viral and Bacterial Diversity during Grass Silage Preservation
Source: Viruses. 2023 Apr 12;15(4):951. doi: 10.3390/v15040951 (PMC10146946; doi:10.3390/v15040951)
Supplement: Supplementary file 1 [file viruses-15-00951-s001.zip › Supplementary_material S1.pdf]

Supplementary material S1

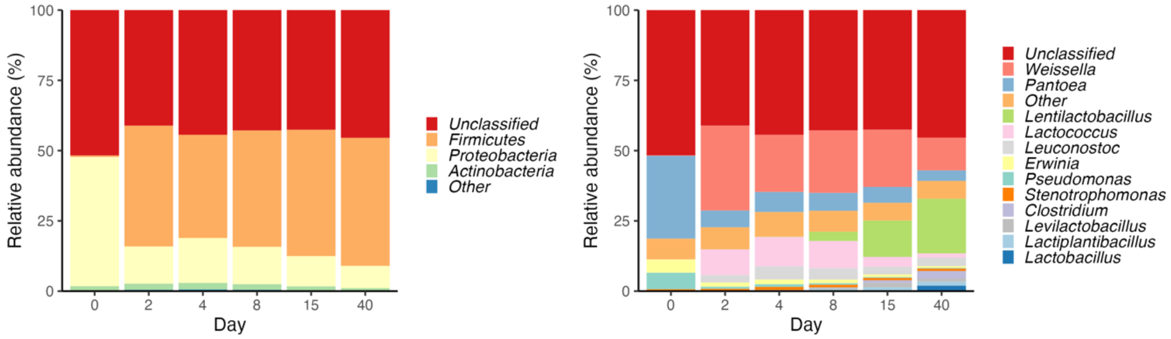

Figure S1. Relative abundance of the most abundant bacterial phyla (left) and genus (right) across 40 days of grass ensilaging. Taxonomic annotation was based on shotgun metagenomic short reads and the NCBI RefSeq database

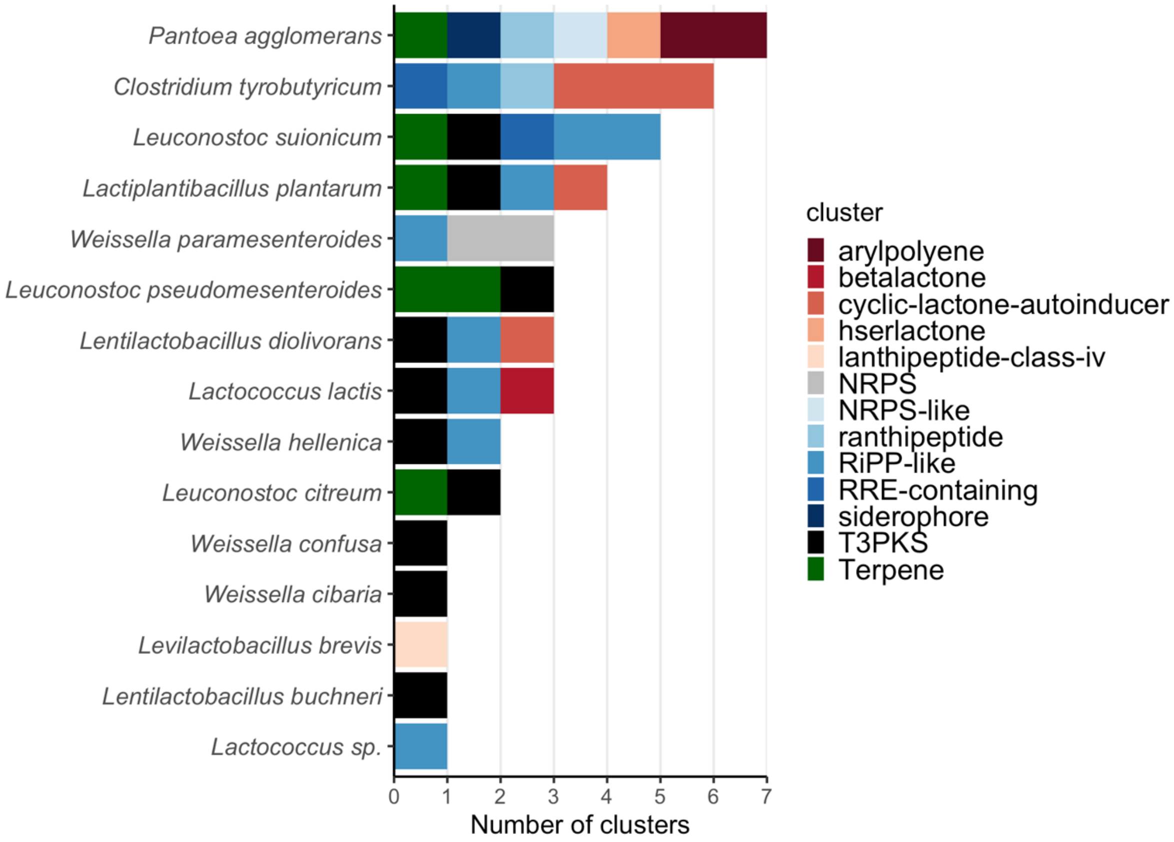

Figure S2. Biosynthetic Gene Clusters (BGC) found in the recovered MAGs from different sampling times across 40 days of grass ensilaging.

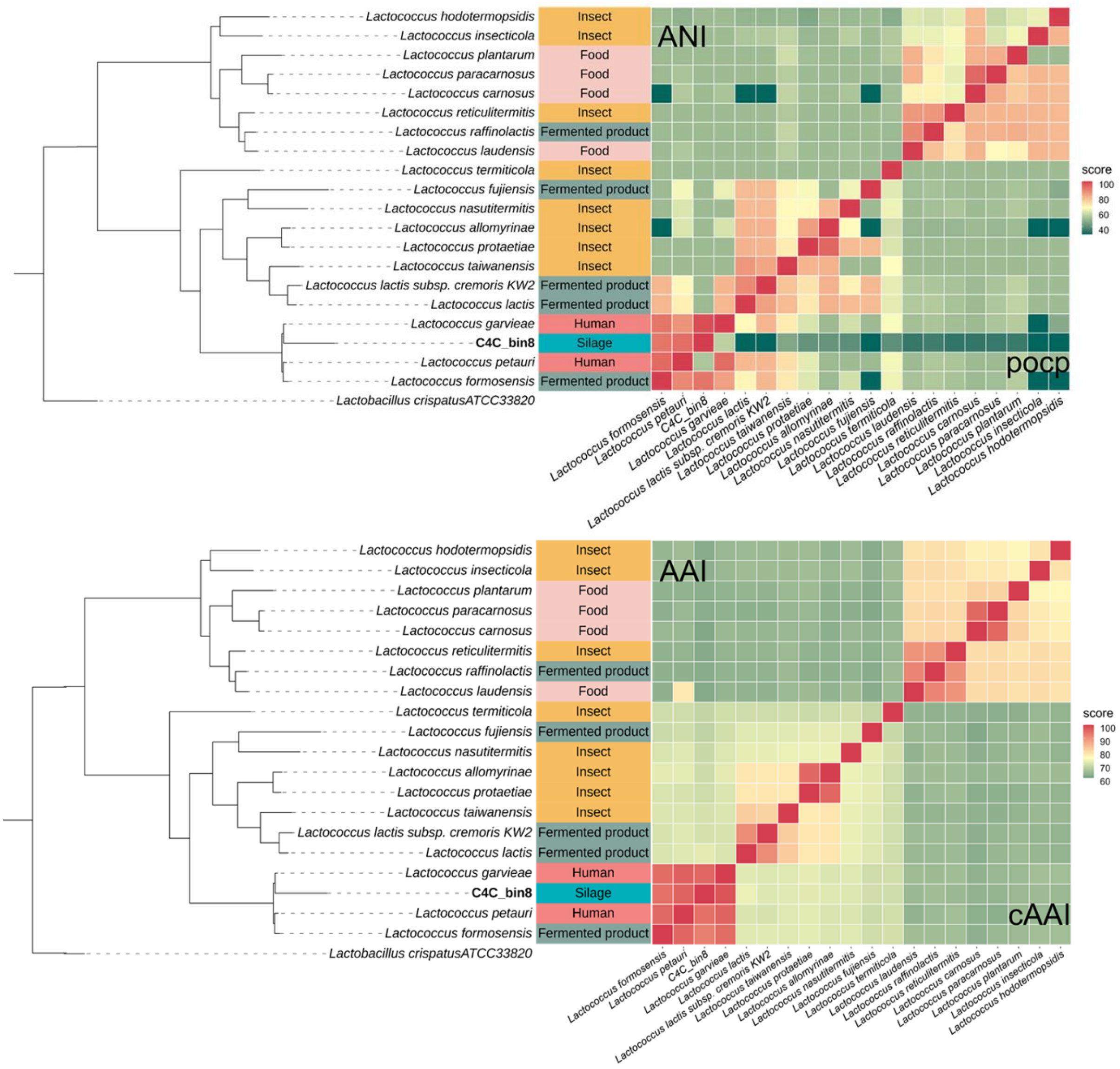

Figure S3. Taxonomic analysis of the MAG C4C.bin.8 using 400 universal bacterial markers and the related identity score index of ANI-pocp (top) and AAI-cAAI (bottom) among members of the genus Lactococcus. The middle stripe depicts the source of isolation from which each genomes originated. Lactobacillus crispatus was used as an external group to root the tree.

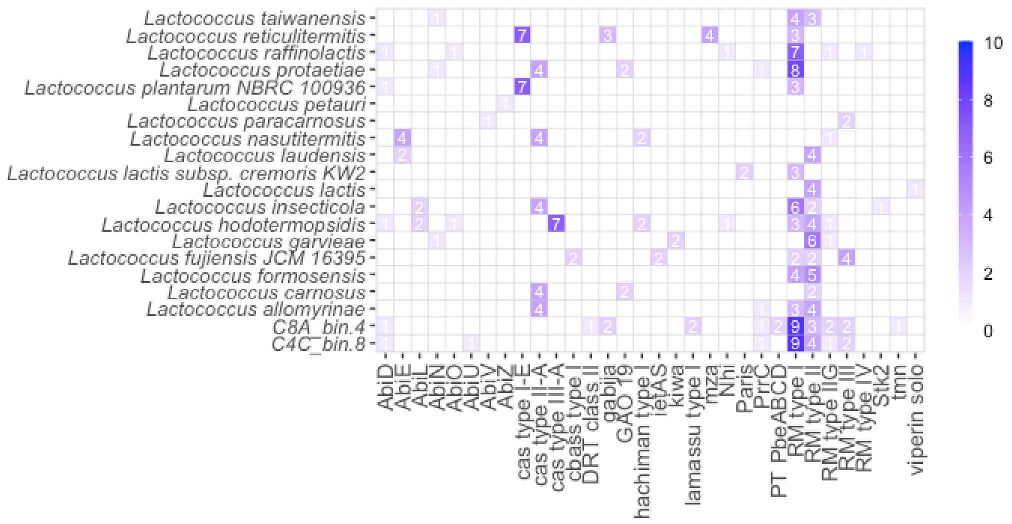

Figure S4. Antiviral defence system found in a set of Lactococcus complete genomes isolated for different sources and 2 MAGs recovered from grass ensilaging samples

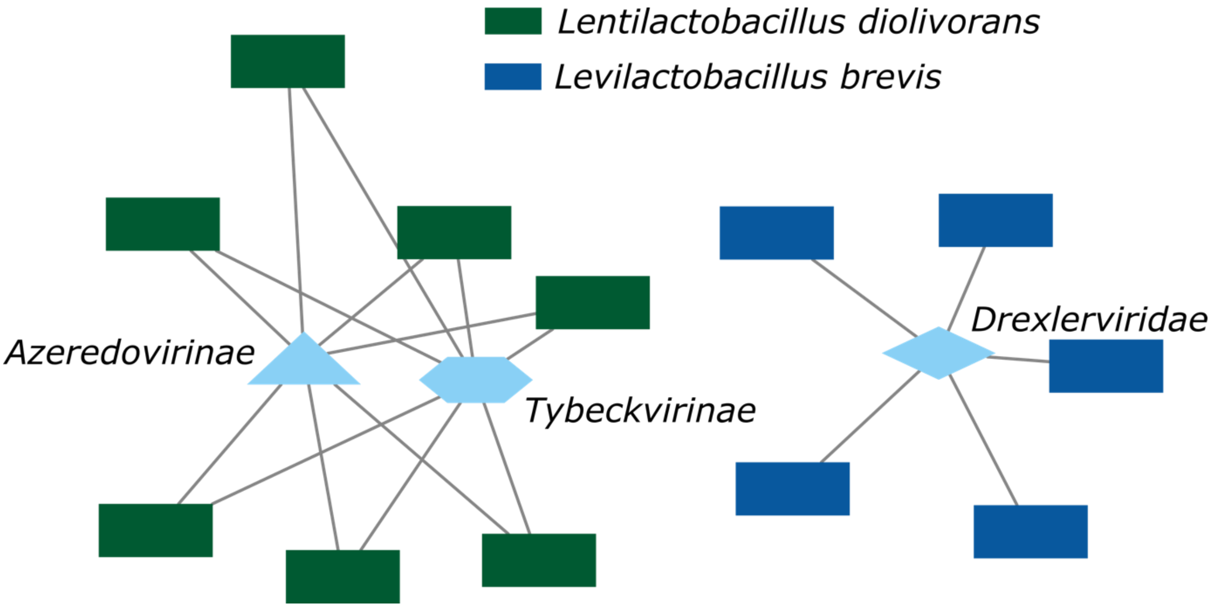

Figure S5. Host virus interaction between bacterial genomes and vOTUs during 40 days of grass ensilaging. The interaction was predicted using the protospacer-to-spacer match. Rectangles indicate different MAGs.

Supplementary methods

qPCR preparation

According to the instructions of the MinElute PCR Purification Kit (Qiagen ,NL) the amplified 16s rRNA was purified from the PCR-Products. To prepare S0 1µL of the sample got diluted with 999µL of nuclease-free water. After this, the standard was gradually diluted 7 times by using 1µL of the standard and 9µL nuclease-free water (dilution 1:10). The qPCR was prepared in a total volume of 20 µL where 1 µL of each sample or standard(S1-S7) was mixed with 10 µL GoTaq® 2x Master Mix (Promega, USA) and 0.2µL of each primer. Programming of the plate and quantification was performed according to manufacturer information (Promega, USA).

Lactococcus pangenome

To obtain the cAAI score, the complete set of genomes of Lactococcus species was analysed as a pangenome using Anvi'o v7.1. All genomes were collected from isolates of Lactococcus available in the databases and pretreated to discard contigs shorter than 2.5 kb that may have originated from low-abundance contaminants. Open reading frame annotation was done using HMMER v3.3.2 and DIAMOND v2.0.15 included in the Anvio Pangenome workflow, to annotate and calculate the similarity of each amino acid sequence across all genomes. The resulting pan-genome output was visualised through the interactive interface of Anvi´o. The core genome calculation was performed considering the presence of 90 % of single-copy genes and an index of geometric homogeneity of 0.95, obtaining 201 genes.
